# Exercise Induced Changes in Salivary and Serum Metabolome in Trained Standardbred, Assessed by ^1^H-NMR

**DOI:** 10.3390/metabo10070298

**Published:** 2020-07-21

**Authors:** Marilena Bazzano, Luca Laghi, Chenglin Zhu, Enrica Lotito, Stefano Sgariglia, Beniamino Tesei, Fulvio Laus

**Affiliations:** 1 School of Biosciences and Veterinary Medicine, University of Camerino, Via Circonvallazione, 93/95, 62024 Matelica, Italy; marilena.bazzano@unicam.it (M.B.); lotitoenrica@gmail.com (E.L.); beniamino.tesei@unicam.it (B.T.); fulvio.laus@unicam.it (F.L.); 2 Department of Agricultural and Food Sciences, University of Bologna, 47521 Cesena, Italy; chenglin.zhu2@unibo.it; 3 Practitioner, 63900 Fermo, Italy; stefano.sgariglia@libero.it

**Keywords:** horse, metabolomic, metabolism, exercise, saliva

## Abstract

In the present study, data related to the metabolomics of saliva and serum in trained standardbred horses are provided for the first time. Metabolomic analysis allows to analyze all the metabolites within selected biofluids, providing a better understanding of biochemistry modifications related to exercise. On the basis of the current advances observed in metabolomic research on human athletes, we aimed to investigate the metabolites’ profile of serum and saliva samples collected from healthy standardbred horses and the relationship with physical exercise. Twelve trained standardbred horses were sampled for blood and saliva before (T_0_) and immediately after (T_1_) standardized exercise. Metabolomic analysis of both samples was performed by ^1^H-NMR spectroscopy. Forty-six metabolites in serum and 62 metabolites in saliva were detected, including alcohols, amino acids, organic acids, carbohydrates and purine derivatives. Twenty-six and 14 metabolites resulted to be significantly changed between T_0_ and T_1_ in serum and saliva, respectively. The findings of 2-hydroxyisobutyrate and 3-hydroxybutyrate in serum and GABA in equine saliva, as well as their modifications following exercise, provide new insights about the physiology of exercise in athletic horses. Glycerol might represent a novel biomarker for fitness evaluation in sport horses.

## 1. Introduction

Metabolic changes related to energy production and cell growth have been observed in response to exercise in humans [1,2,3,4]. Although these studies have contributed to the understanding of the metabolic response after physical exercise, the limited number of metabolites taken into account does not represent the broader metabolic response caused by exercise [2,4,5]. Human serum accounts for about 4000 metabolites [6] that interact in a large and complex network [7]. Therefore, utilizing a more efficient and comprehensive method to analyze all the metabolites within selected biofluids could provide a better understanding of the metabolic response in the context of biochemistry modifications related to exercise.

In this context, the employment of metabolomic analysis can be essential, as demonstrated in a study by Berton and colleagues (2017) [8] that focused on the possibility of using serum as a valuable biofluid to investigate metabolomic modifications induced by exercise in sports players [8].

However, serum is only one of the biofluids suitable for scientific research in the field of sport medicine. A special interest was recently pointed towards the use of saliva as a diagnostic fluid both in human and veterinary medicine [9,10]. It is well known that saliva composition can be affected by systemic disorders and may reflect general metabolic changes [11]. When compared with other biological samples, saliva has the advantage of being easily collected by non-invasive and non-stressful procedures, which is extremely important when sampling animals [9]. The composition of saliva is 99% water but it also contains several compounds such as hormones, glucose, lactate, fatty acids, triglycerides, cholesterol, urea, uric acid and phosphorus [9]. Metabolites, enzymes, proteins and minerals have been found in saliva samples collected from humans, pigs, sheep and horses [9,12,13,14,15,16,17]. Some of these metabolites have been found to change as a result of pathological conditions like lameness, stress, abdominal pain, inflammation and kidney diseases, and could be eligible as biomarkers in humans and animals [9,12,13,16,18,19]. A proteomic approach was also applied to saliva samples from humans, horses, cattle, dogs, sheep, rabbits and rats with the aim to establish specific proteome signatures of mammals’ saliva [20]. Recent studies focused on the possibility of using saliva as a valuable biofluid to investigate enzymatic [17] and metabolomic [21] modifications induced by exercise in soccer players. Exercise testing and monitoring of training sessions have an important value in the assessment of poor performance, fitness and performance potential in athletic horses [22,23,24]. However, despite a continuous search for novel methods to evaluate the equine athlete, little is known about its metabolomic profile [25,26], and no information is available on the metabolome of standardbred horses

On the basis of the current advances observed in metabolomic research, we aimed to investigate the metabolomic profile of serum and saliva samples collected from healthy standardbred horses and the relationship with physical exercise. Closely monitoring fitness, workload and injuries in sport horses is a major matter to better understand the effects of training methods, so to reduce injuries [24]. That is why the evaluation of indices of fitness (starting from blood parameter, velocity and/or heart rate) is important to adapt training programs with the double aim to improve performance and preserve horses’ welfare [24]. Metabolomics applied to athletes could provide new insight in the adaption of horses to exercise, evaluating in detail which metabolites are altered, trying to give an explanation and apply any practical corrective measures. Among the high-throughput platforms employed for metabolomics, we decided to employ proton nuclear magnetic resonance (^1^H-NMR) spectroscopy, that ensures data traceability, reproducibility and interoperability, because the only variables modulating an NMR spectrum are magnetic field, solvent and pulse sequence [27].

## 2. Results

All the horses included in the study were accustomed to the training program and showed no clinical sign of disease during the experimental period. The saliva collection method herein used was non-invasive and well tolerated by all horses. 

**Figure 1:**
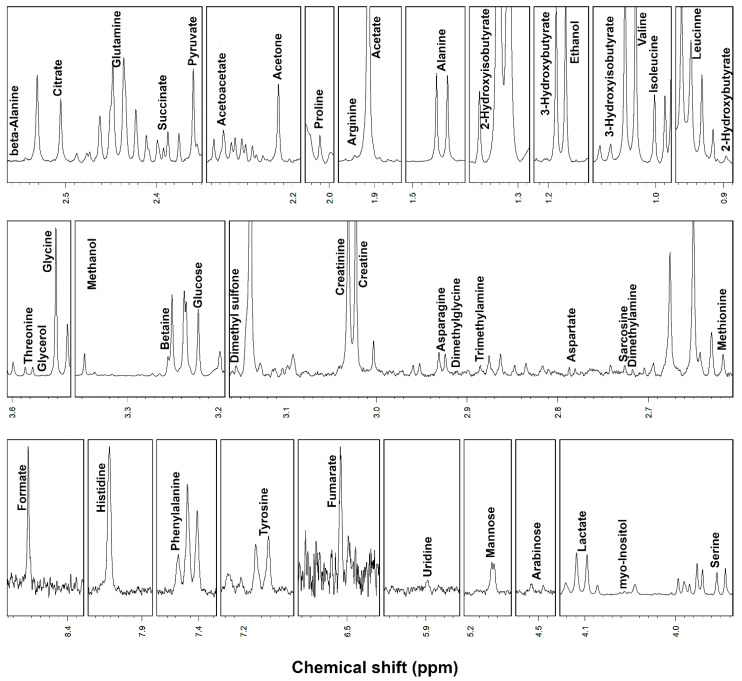
Portions of ^1^H-NMR spectra from typical serum samples. Assignments appear on the signals used for molecules quantification. The vertical scale of each portion is conveniently set to ease the signals observation.

**Figure 2:**
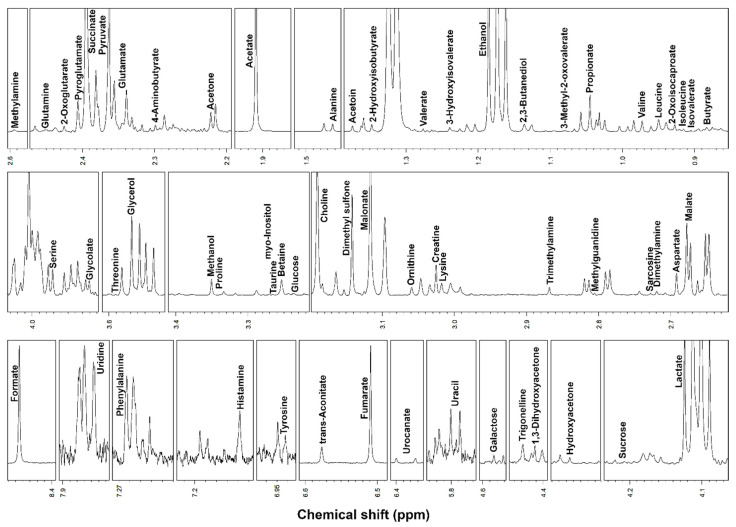
Portions of ^1^H-NMR spectra from typical saliva samples. Assignments appear on the signals used for molecules quantification. The vertical scale of each portion is conveniently set to ease the signals observation.

^1^H-NMR spectroscopy allowed to quantify 46 metabolites in serum and 62 metabolites in saliva, including, among others, alcohols, amino acids, organic acids, carbohydrates and purine derivatives (Figure 1 and Figure 2), with a spectrum of substances broadly in line with those found in the human metabolome [8,28].

Despite the detection of 30 shared metabolites, serum and salivary metabolomes resulted to be different, with 16 metabolites found only in serum, and 32 metabolites found only in saliva.

The concentrations of the metabolites that resulted to be statistically different between the samples collected before (T_0_) and after (T_1_) exercise in serum are reported in Table 1. *p*-values for all non-significant metabolites are reported in Supplementary Materials Table S1.

**Figure 3:**
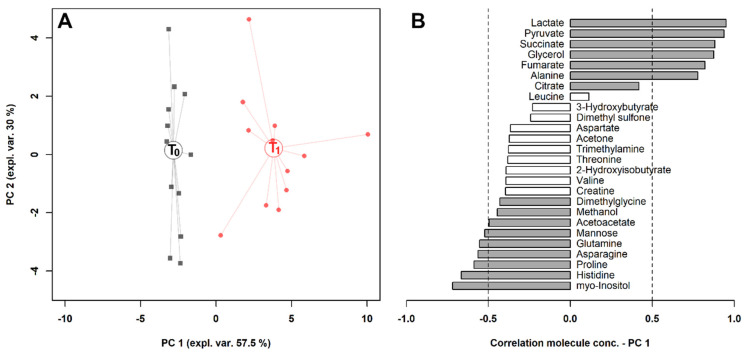
rPCA model built on the space constituted by the concentration of the molecules significantly different in serum, listed in Table 1. (**A**) In the score plot, samples collected at T_0_ and T_1_ are represented with black squares and red circles, respectively. The wide, empty circles represent the median of the groups. (**B**) The loading plot reports the correlation between the concentration of each substance and its importance over principal component (PC) 1. Significant correlations (*p* < 0.05) are highlighted with gray bars.

To observe the overall variations intrinsic to the samples in the space constituted by this restricted group of metabolites, we calculated on their concentrations a robust Principal Component Analysis (rPCA) model (Figure 3). Three principal components (PCs) were accepted, the first of which accounted for 57.5% of the samples’ variance represented by the model. Such a PC nicely accounted for the differences among the samples connected to exercise and showed that lactate, pyruvate, succinate, glycerol, fumarate and alanine mostly characterized the samples collected after exercise, while myo-inositol, histidine, proline, asparagine, glutamine and mannose mostly characterized the samples collected before exercise.

About the salivary metabolome, the concentrations of five molecules significantly increased after exercise, while nine significantly decreased between T_0_ and T_1_ (Table 2). *p*-values for all non-significant metabolites are reported in Supplementary Materials Table S2.

**Figure 4:**
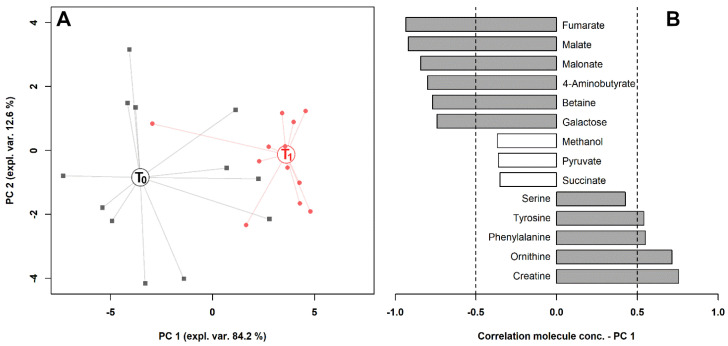
rPCA model built on the space constituted by the concentration of the molecules significantly different in saliva, listed in Table 2. (**A**) In the score plot, samples collected at T_0_ and T_1_ are represented with black squares and red circles, respectively. The wide, empty circles represent the median of the groups. (**B**) The loading plot reports the correlation between the concentration of each substance and its importance over PC 1. Significant correlations (*p* < 0.05) are highlighted with gray bars.

**Figure 5:**
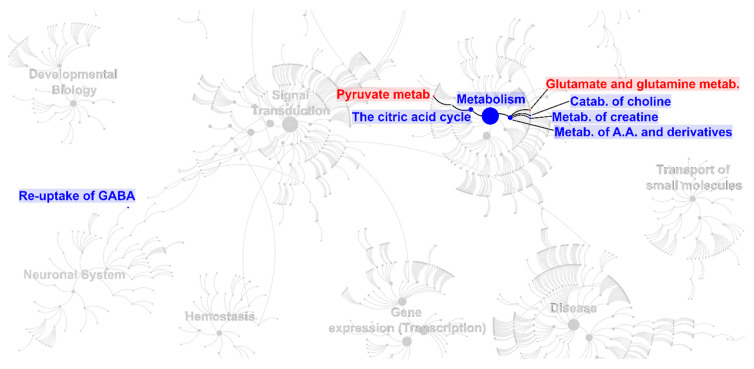
Biomolecular pathways overrepresentation analysis performed on the molecules listed in Table 1 for serum (red) and Table 2 for saliva (blue). The figure replicates a convenient portion of the biomolecular pathways overview according to Reactome, modified to show the main pathways and sub-pathways overrepresented as a consequence of exercise.

To observe the overall trends associated with the samples, we calculated on their concentrations the rPCA model outlined in Figure 4. Three principal components (PCs) were accepted, the first of which accounted for 84.2% of the samples’ variance represented by the model. Such PCs summarized the differences among the samples connected to exercise. The salivary metabolome of horses after exercise was mainly characterized by creatine, ornithine, phenylalanine and tyrosine, while horses before exercise were mainly characterized by fumarate, malate, malonate, 4-aminobutyrate, betaine and galactose. In Figure 5, the metabolic pathways overrepresented by the molecules significantly affected by exercise in serum and saliva are reported.

## 3. Discussion

The results showed in the present paper highlight for the first time the changes occurring in the metabolomic profile of trained standardbred horses following exercise in both serum and saliva specimens.

Biochemical constituents of the tricarboxylic acid cycle (TCA or Krebs cycle) including succinate and fumarate were significantly increased in serum after exercise. This is in agreement with results obtained by other authors in human and equine athletes [25,29,30] and could be explained by the need to maintain and/or increase the Krebs cycle flow during exercise [8]. On the contrary, succinate and fumarate, together with malate, another constituent of the TCA cycle, have been found to decrease in saliva after exercise. Among salivary metabolites, malonate was found to have a very strong inhibitory effect on the TCA cycle in complex metabolic systems. Malonate is not normally present in cells [31] and the reason for its presence in saliva, its decrease after exercise and its inhibitory effect on the TCA cycle in horses during exercise need further investigations. Notwithstanding the paucity of data on salivary metabolomics during exercise, we can speculate that the decreased salivary level of succinate, fumarate and malate might reflect the imbalance of the aerobic pathways in favor of the anaerobic production of energy during exercise. 

The increase in pyruvate and lactate serum concentrations was expected, since it reflects the anaerobic pathway activation during exercise [8,25]. The high energy demand required during exercise can overload the mitochondria’s ability to oxidize pyruvate that is converted to lactate, supplying energy by the Cori cycle [8]. In our study, serum lactate increased up to 11.6-fold following exercise, and this result was beyond the physiological range usually reported for human athletes of a 9.3–9.6-fold increase [8,32]. Probably, the greater muscle mass of horses with respect to humans can account for this difference. Although not significant, pyruvate and lactate slightly decreased in saliva after exercise (Supplemental Materials Table S1), demonstrating once again that salivary metabolic changes do not follow the same metabolic changes observed in serum, at least regarding the energy supply.

Among the metabolites detected in plasma, there are also two carbohydrates: mannose and arabinose.

The concentration of mannose resulted more concentrated in post-exercise equine plasma. This monosaccharide is found in small amounts in the diet, and after conversion to fructose-6-phosphate, it can be used in both glycolysis and gluconeogenesis [33]. Unlike mannose, the concentration of arabinose in serum appeared as decreased by exercise. Arabinose is known to have an inhibitory effect on intestinal sucrase [34], but its role in horse during exercise in not clear.

Galactose was the only carbohydrate with a changed concentration in saliva, as it decreased at T_1_. In a study on soccer players, Pitti et al. [21] found an increased level of galactose in blood after exercise. Galactose is an important source of glucose and its role in liver glycogen restoration after exercise has been well recognized in cyclists [33,35]. The reason for galactose reduction in the saliva of horses could reflect a different energetic role of this metabolite compared with humans.

Among amino acids, alanine and histidine increased significantly after exercise. Serum alanine increased 1.6-fold soon after exercise, in agreement with the results obtained in humans by Berton et al. [8] but in contrast with Nieman et al. [29]. Alanine in muscle derives from pyruvate, in turn originated from glucose breakdown [8]. This metabolic mechanism is in line with the rise in serum pyruvate found in our study. Furthermore, alanine has a detoxing function by transporting to the liver the large amount of ammonia typically produced during short and high-intensity exercise derived from branched chain amino acids [8,36]. Similarly, the increase in gluconeogenic amino acid histidine could be linked to the higher energetic demand during acute exercise. 

The decreased levels of glutamine, asparagine and proline observed in horses after training were similar to the findings in human athletes [29,30] thus supporting the hypothesis of an enhanced muscle amino acid oxidation during exercise to sustain the energetic demand.

In equine saliva, amino acids like tyrosine and phenylalanine rose almost 2-fold after exercise. Tyrosine results from hydroxylation of phenylalanine, an essential amino acid [37], and it is metabolized to acetoacetate and fumarate or used as a precursor of catecholamines that play an important role in athletic performance during exercise [38]. We can speculate that the rise in salivary tyrosine after exercise could reflect an increased production to encounter the need for catecholamines during exercise, but the lack of similar changes in serum needs to be clarified. 

Sarcosine (N-methylglycine) is a non-proteinogenic amino acid derivative and occurs in the body as a product of the metabolism of glycine and creatine [39]. In addition to its multiple functions in the body and its use as a potential marker in various diseases, it has been an ingredient in toothpaste for decades as it prevents tooth decay and causes foaming [39]. The reason for the slight rising of sarcosine and creatine in equine saliva after exercise is not clear, but its physiological function could be protective for teeth health.

Ornithine levels increased up to 2.1-fold in saliva but not in serum, differently from what has been observed in human athletes where it is down regulated 60–70 min after exercise as a consequence of the accelerated urea cycle due to the higher production of ammonia [8,30]. However, the differences in the sampling times of our study compared with others might explain this discrepancy. Betaine, a small molecule acting as a methyl donor in minor pathways [37], decreased in saliva after exercise, but this finding needs further investigations.

The marked elevation in serum glycerol in our horses following exercise resulted from an extensive lipolysis and is consistent with the study by Lewis et al. [30], who found higher serum glycerol up-regulation after exercise in fitter athletes. Considering that all the horses included in our study were well trained, we could consider glycerol a marker of adaptation to training also in equine species.

Salivary methanol was reduced 1.8 times by exercise. The same result was recently found in the saliva of soccer players, and the change was ascribed to the higher evaporation of volatile compounds in the mouth during exercise [21]. In a recent study, methanol was considered a marker of inflammation in horses affected by equine asthma when detected in exhaled breath condensate (EBC) [40].

Uracil nucleotide, together with cytosine, are the major pyrimidine components of RNA. Uracil can be utilized in glycogen synthesis [41] and its rise in saliva could be related to the higher energetic demand during exercise.

2-hydroxyisobutyrate was up-regulated in the serum of standardbred horses following exercise. This metabolite is a normal constituent of human serum and saliva [42] that has been proposed as a biomarker for glycogen storage disease type 1a in juveniles and acute coronary syndrome [43,44]. It was also found decreased in plasma of Alzheimer’s disease patients vs. controls [45], however no information is available about its role during sport activity.

3-hydroxybutyrate decreased in serum after exercise. An elevation of this ketone was documented in marathon runners due to ketone production [30]. It was also found to be increased in runners after a three-day intensified exercise as a result of fatty acid oxidation [29]. On the other hand, Berton et al. [8] did not find any change in 3-hydroxybutyrate after a leg press resistance exercise. Our result might suggest that this metabolic pathway is of negligible importance in trained standardbred horses performing acute exercise of short duration.

4-aminobutyrate (γ-Aminobutiryc acid or GABA) is the most inhibitory neurotransmitter in the central nervous system [46] and its presence has been already demonstrated in human saliva [47] as well as in salivary glands of men and rats [48,49], being associated with its biosynthetic and metabolic enzymes. However, this is the first report showing the presence of GABA in equine saliva and the down-regulatory effect of exercise in this species. GABA and its receptors can be found in other non-neuronal organs but its role in peripheral tissue remains to be established, although it is known to be involved in cellular proliferation [48]. Authors suggested a role of GABA(A)-R in the suppression of salivary secretion in rat salivary glands [50]. Similarly, the down-regulation of salivary GABA during exercise in horses could be associated with this regulatory function of saliva secretion, possibly linked to thermoregulation.

Significantly lower levels of myo-inositol have been found both in the serum and saliva of horses after exercise. Myo-inositol promotes the maturation of pulmonary surfactants and supports respiratory function [51], modulates cytoskeleton dynamics, thus allowing alveolar cells to counteract collapsing forces and promoting mechanical stabilization of cell shape [52], and it recruits water and organic compounds in the alveolar space, decreasing surface tension through the formation of a biofilm layer at the interface [53]. Myo-inositol is also the most effective allosteric effector identified to date, being able to increase the tissue delivering of oxygen bound to hemoglobin [54]. Since its administration can improve sport performance in laboratory mice, its analogues have been suspected to be abused in the horse racing industry [55]. Up to now, no study about its effect in equine species has been performed. Recently, reduced concentrations of myo-inositol have been found in the bronchoalveolar lavage fluid of horses affected by equine asthma in comparison with healthy horses [56]. The exact role of this metabolite in physical exercise is not known, however, the decrease observed in both serum and saliva could indicate an impairment in the normal respiratory function during exercise. 

## 4. Materials and Methods

### 4.1. Animals

Twelve clinically healthy standardbred horses (7 males and 5 females), mean age 6.7 years old, mean body condition score 3 out of 5 [57], were included in the study with informed owner consent. 

All horses were stabled in individual boxes and were fed the same polyphyte hay at a ratio of 2 kg/100 kg BW, and commercial horse feed in the amount of 0.8 kg/100 kg of BW (protein 11%; fat 4%; fiber 8.56%; Ca/P 2:1) twice a day (at 10 am and at 5 pm). Clean potable drinking water was offered ad libitum. 

The sampling herein described was part of a normal procedure for monitoring the training status of the animals. All procedures were approved by the Animal Care Committee of Camerino University (Registration number E81AC.10/Ac) and were in accordance with the standard recommended by the EU Directive 2010/63/EU for experimental animals.

### 4.2. Experimental Procedure 

All horses were trained 6 days per week and the training program consisted of 7 runs of 1000 m each at different speed. Briefly, the first warm-up run clockwise at light trot (mean speed 5 m/s) was followed by 3 counter clockwise runs (mean speed 10 m/s), then 1 recovery run at walking and 2 runs at full speed trot (mean speed 12 m/min), and finally 2 runs at light trot (mean speed 5 m/s). All animals were sampled the day before the rest-day.

Saliva and blood samples were collected before (T_0_) and immediately after full speed exercise (T_1_). Saliva samples were collected by using cotton swabs and Salivette^®^ tubes (Sarstedt AG & Co., Nümbrecht, Germany). The cotton swab was grasped with a surgical clamp, inserted at the angle of the lips into the mouth of the horse and placed gently on the tongue surface for 5 min and then inserted into the Salivette^®^ tube. 

Venous blood samples were collected at T_0_ and T_1_ by jugular venepuncture into 4 mL vacutainer sterile tubes containing EDTA (Vacuette^®^ Greiner Bio-One, Cassina de Pecchi, Italy) and 9 mL vacutainer sterile tubes containing clot activators (Vacuette^®^ Greiner Bio-One, Cassina de Pecchi, Italy). A complete blood count (CBC) and biochemical profile was performed on blood and serum samples to exclude systemic disorders.

Whole blood was directly tested in the field for blood lactate concentration by using a portable device (Accutrend Plus^®^, Roche, Mannheim, Germany) at T_0_ and T_1_ as a part of the fitness evaluation routine.

All the sampling procedures were performed before feeding, at the same time of the day (06:00–8:00 a.m.) and, immediately after collection, all the samples were stored at 5 °C and delivered to the lab within 2 h.

### 4.3. Laboratory Analysis. 

Saliva samples were obtained by centrifugation of Salivette^®^ tubes (10 min at 1000 g) (Universal 32, Hettich Zentrifugen, Tuttlingen, Germany) and 2 mL aliquots of supernatant were stored at −20 °C until metabolomic analysis.

Blood samples with clot activators were centrifuged for 10 min at 1000 g (Universal 32, Hettich Zentrifugen, Tuttlingen, Germany) and the obtained sera were stored at −20 °C until analysis.

For ^1^H-NMR analysis, we created a stock solution composed of 3-(trimethylsilyl)-propionic-2,2,3,3-d4 acid sodium salt (TSP) 10 mmol/L and NaN_3_ 2 mmol/L in D_2_O. The former served as the NMR spectra chemical-shift reference, while the latter avoided bacteria proliferation. The solution was set to pH at 7.00 ± 0.02 by phosphate buffer (1 M). Both serum and saliva samples were prepared for ^1^H-NMR by thawing and centrifuging 1 mL of each sample at 4 °C for 15 min at 18,630 g. The supernatant (700 μL) was added to 100 μL of the NMR analysis solution and centrifuged again. 

The spectra were recorded with an AVANCE III spectrometer (Bruker, Milan, Italy), controlled by the Topspin software (Ver. 3.5), at a frequency of 600.13 MHz and a temperature of 298 K. The residual signal from the water was suppressed by pre-saturation, while broad signals from large molecules were reduced by a CPMG-filter, set as outlined by Zhu et al. [58]. Each spectrum was acquired by summing up 256 transients registering 32 K data points over a 7184 Hz spectral window, with an acquisition time of 2.28 s and relaxation delay of 5 s.

In Topspin, a manual correction phase was applied to each spectrum, together with a line-broadening of 0.3 Hz. The subsequent steps were performed in R computational language by means of scripts developed in-house. The spectra were aligned toward the right peak of the alanine doublet, set to 1.473 ppm. Baseline was then corrected, after having removed the residual water signal, by isolating irregularities of the baseline by peak detection, according to the “rolling ball” principle [59].

For signals’ assignment, chemical shift and multiplicity were compared with the Chenomx software library (Chenomx Inc., Edmonton, AB, Canada, ver 8.3). The added TSP was employed as internal standard in the first sample analyzed. Differences in water content among samples were then taken into consideration by probabilistic quotient normalization [27]. Rectangular integration was employed to quantify each molecule, by focusing on one signal per molecule free from superimpositions.

### 4.4. Statistical Analysis

Differences between the two groups were looked for by means of a paired *t*-test applied to the concentrations of each molecule, transformed by the Box-Cox algorithm for normal distribution [60].

We highlighted any trend characterizing the samples with robust principal component analysis (rPCA) models [61], based on the molecules accepted by the univariate analysis. To this purpose, we employed the PcaHubert algorithm implemented in the rrcov package. The main features of each rPCA model are summarized by a score plot and by a Pearson correlation plot. The former is the projection of the samples in the PC space and highlights the underlying structure of the data. The latter relates the concentration of each variable to the components of the model.

Metabolic pathway analysis was conducted by relying on the Reactome pathway knowledgebase [62], with overrepresentation analysis (ORA) based on a hypergeometric test [63].

## 5. Conclusions

In the present study, data related to the metabolomics of saliva and serum in trained standardbred horses have been provided for the first time. Several molecules are common to metabolic pathways already described in human athletes (e.g., energetic metabolism). However, the findings of 2-hydroxyisobutyrate and 3- hydroxybutyrate in serum and GABA in equine saliva, as well as their modifications following exercise, provide new insights about the physiology of exercise in athletic horses. Furthermore, the use of metabolomic analysis allowed to identify metabolites like glycerol that might represent novel biomarkers for fitness evaluation in sport horses. However, further investigations are needed to validate the metabolites herein found as indices of fitness levels in equine species.

## Figures and Tables

**Table 1:** Serum metabolites with significantly different concentration (μmol/L; mean ± SD) before (T_0_) and after (T_1_) exercise.

Molecule	T_0_	T_1_	*p*	Trend *
3-Hydroxybutyrate	2.09 ± 1.57	1.52 ± 1.33	4.58 × 10^−2^	↓
2-Hydroxyisobutyrate	1.64 ± 0.96	0.95 ± 1.19	6.19 × 10^−5^	↓
Acetoacetate	3.73 ± 0.73	3.21 ± 0.58	3.20 × 10^−4^	↓
Acetone	3.31 ± 1.42	2.56 ± 0.85	1.45 × 10^−2^	↓
Alanine	66.34 ± 10.37	104.87 ± 11.85	4.87 × 10^−8^	↑
Asparagine	10.36 ± 1.93	7.44 ± 1.82	7.92 × 10^−5^	↓
Aspartate	2.17 ± 1.17	1.53 ± 0.61	2.23 × 10^−2^	↓
Citrate	11.24 ± 5.88	16.40 ± 7.51	1.79 × 10^−2^	↑
Creatine	22.76 ± 7.67	17.94 ± 5.91	2.95 × 10^−4^	↓
Dimethyl sulfone	14.74 ± 8.56	11.09 ± 5.96	4.32 × 10^−3^	↓
Dimethylglycine	0.36 ± 0.13	0.28 ± 0.09	3.91 × 10^−3^	↓
Fumarate	0.84 ± 0.21	1.50 ± 0.73	8.82 × 10^−3^	↑
Glutamine	71.51 ± 11.48	58.14 ± 6.78	6.18 × 10^−5^	↓
Glycerol	4.03 ± 3.01	45.51 ± 22.46	1.84 × 10^−5^	↑
Histidine	29.91 ± 4.34	23.46 ± 3.35	1.52 × 10^−3^	↓
Lactate	141.26 ± 50.20	1143.45 ± 582.63	1.03 × 10^−4^	↑
Leucine	35.68 ± 6.37	39.22 ± 6.87	9.62 × 10^−3^	↑
Mannose	11.20 ± 2.71	7.85 ± 2.09	1.33 × 10^−5^	↓
Methanol	16.98 ± 10.21	10.26 ± 8.25	1.49 × 10^−3^	↓
myo-Inositol	10.35 ± 1.68	7.37 ± 2.06	8.38 × 10^−4^	↓
Proline	10.57 ± 2.45	8.02 ± 1.54	4.19 × 10^−4^	↓
Pyruvate	8.72 ± 2.37	26.61 ± 7.59	4.38 × 10^−6^	↑
Succinate	1.12 ± 0.21	4.24 ± 1.79	6.11 × 10^−5^	↑
Threonine	37.30 ± 5.45	33.26 ± 6.98	4.93 × 10^−3^	↓
Trimethylamine	0.16 ± 0.05	0.14 ± 0.05	2.73 × 10^−3^	↓
Valine	54.09 ± 6.79	50.40 ± 9.04	1.85 × 10^−2^	↓

* Increasing (↑) or decreasing (↓) trends from T_0_ to T_1_.

**Table 2:** Salivary metabolites with different concentrations (μmol/L; mean ± SD) before (T_0_) and after (T_1_) exercise.

Molecule	T_0_	T_1_	*p*	Trend *
4-Aminobutyrate	30.09 ± 15.39	10.87 ± 16.55	4.35 × 10^−3^	↓
Betaine	143.58 ± 86.06	67.19 ± 52.51	1.23 × 10^−2^	↓
Creatine	36.68 ± 19.11	62.11 ± 26.59	1.28 × 10^−2^	↑
Fumarate	94.70 ± 58.46	23.20 ± 29.32	8.35 × 10^−4^	↓
Galactose	21.68 ± 10.18	12.55 ± 12.66	1.98 × 10^−2^	↓
Malate	550.72 ± 395.87	138.69 ± 166.50	2.11 × 10^−3^	↓
Malonate	193.93 ± 107.17	46.84 ± 35.06	5.10 × 10^−4^	↓
Methanol	131.26 ± 73.70	73.05 ± 50.01	5.07 × 10^−3^	↓
Ornithine	22.36 ± 12.53	46.91 ± 21.66	3.24 × 10^−3^	↑
Phenylalanine	28.22 ± 11.81	53.82 ± 23.36	6.10 × 10^−3^	↑
Pyruvate	70.77 ± 32.43	49.34 ± 24.69	7.41 × 10^−2^	↓
Sarcosine	2.65 ± 1.64	4.48 ± 2.94	2.48 × 10^−2^	↑
Succinate	210.91 ± 78.35	120.47 ± 80.66	3.59 × 10^−2^	↓
Tyrosine	33.32 ± 18.13	66.07 ± 46.48	3.29 × 10^−2^	↑

* Increasing (↑) or decreasing (↓) trends from T_0_ to T_1_.

## Supplementary Materials

The following are available online at https://www.mdpi.com/2218-1989/10/7/298/s1, Table S1: Serum metabolites with no significant difference in concentrations before and after exercise (µmol/L; mean ± SD), Table S2: Salivary metabolites with no significant difference in concentrations before and after exercise (µmol/L; mean ± SD).
