# Supplementary material for: Exercise Induced Changes in Salivary and Serum Metabolome in Trained Standardbred, Assessed by 1H-NMR
Source: Metabolites. 2020 Jul 21;10(7):298. doi: 10.3390/metabo10070298 (PMC7407172; doi:10.3390/metabo10070298)
Supplement: Supplementary file 1 [file metabolites-10-00298-s001.pdf]

Exercise induced changes in salivary and serum metabolomic metabolome in trained standardbred, assessed by ^{1}H-NMR

Supplemental material

Table S1: Serum metabolites with no significant difference in concentrations before and after exercise (µmol/L; mean ± SD).

	T_0	T_1	*p*
Formate	4.17 ± 0.88	3.62 ± 0.73	0.07
Phenylalanine	16.75 ± 3.27	15.66 ± 2.68	0.17
Tyrosine	12.53 ± 3.81	10.80 ± 1.71	0.11
Uridine	4.29 ± 0.63	4.09 ± 1.07	0.59
Arabinose	9.49 ± 1.51	8.23 ± 0.94	0.05
Serine	40.07 ± 13.64	38.76 ± 11.65	0.64
Glycine	124.75 ± 35.40	132.87 ± 36.27	0.25
Betaine	4.30 ± 1.64	4.57 ± 1.43	0.50
Glucose	642.24 ± 67.69	674.23 ± 97.82	0.24
Creatinine	12.47 ± 2.46	11.16 ± 3.17	0.20
Sarcosine	0.56 ± 0.20	0.50 ± 0.17	0.25
Dimethylamine	0.18 ± 0.08	0.18 ± 0.06	0.89
Methionine	6.78 ± 1.36	5.80 ± 1.10	0.07
Beta-Alanine	2.17 ± 0.77	1.79 ± 0.65	0.12
Arginine	26.00 ± 10.19	22.57 ± 5.17	0.22
Acetate	107.31 ± 67.92	93.07 ± 34.07	0.29
2-Hydroxybutyrate	136.91 ± 346.91	18.57 ± 6.99	0.26
Ethanol	491.69 ± 1565.43	0.76 ± 1.00	0.30
3-Hydroxyisobutyrate	5.67 ± 3.56	4.47 ± 1.40	0.17
Isoleucine	12.72 ± 1.55	11.82 ± 1.83	0.07

Table S2: Salivary metabolites with no significant difference in concentrations before and after exercise (µmol/L; mean ± SD).

	T_0	T_1	*p*
1,3-Dihydroxyacetone	4.19 ± 1.45	5.48 ± 3.54	0.23
2,3-Butanediol	12.22 ± 5.05	10.91 ± 9.74	0.70
2-Hydroxyisobutyrate	7.58 ± 4.81	4.74 ± 4.07	0.14
2-Oxoglutarate	14.13 ± 5.93	14.12 ± 7.69	1.00
2-Oxoisocaproate	5.40 ± 2.31	6.05 ± 1.65	0.48
3-hydroxyisovalerate	6.30 ± 9.79	3.95 ± 7.13	0.51
3-Methyl-2-oxovalerate	3.88 ± 1.85	5.08 ± 7.25	0.60
Acetate	2185.19 ± 1367.95	2445.27 ± 964.39	0.60
Acetoin	6.94 ± 5.62	6.37 ± 8.39	0.86

Acetone	5.54 ± 3.19	3.96 ± 2.05	0.19
Alanine	123.83 ± 45.39	117.81 ± 44.08	0.61
Aspartate	28.45 ± 20.30	18.01 ± 14.43	0.13
Butyrate	24.71 ± 9.00	27.66 ± 16.93	0.60
Choline	59.57 ± 12.48	48.23 ± 24.31	0.19
Dimethyl sulfone	43.19 ± 43.72	45.25 ± 37.14	0.70
Dimethylamine	1.85 ± 0.88	7.66 ± 20.89	0.36
Ethanol	580.01 ± 1301.87	1616.19 ± 4507.75	0.47
Formate	110.00 ± 49.10	111.22 ± 72.76	0.96
Glucose	630.52 ± 559.92	365.43 ± 396.59	0.12
Glutamate	55.18 ± 24.55	57.12 ± 21.30	0.64
Glutamine	12.64 ± 6.59	24.49 ± 26.45	0.18
Glycerol	11222.05 ± 5298.82	11190.73 ± 4916.59	0.98
Glycolate	41.39 ± 13.79	54.44 ± 19.11	0.08
Histamine	20.83 ± 10.57	30.61 ± 26.57	0.31
Hydroxyacetone	5.27 ± 3.14	4.38 ± 6.23	0.62
Isoleucine	23.47 ± 8.30	27.05 ± 8.95	0.26
Isovalerate	6.64 ± 8.55	7.65 ± 6.43	0.53
Lactate	1290.02 ± 773.99	938.65 ± 744.91	0.26
Leucine	33.06 ± 15.25	41.22 ± 16.98	0.20
Lysine	59.32 ± 20.58	64.99 ± 20.82	0.55
Methylamine	3.98 ± 2.00	4.27 ± 4.81	0.87
Methylguanidine	1.63 ± 0.49	2.03 ± 2.03	0.53
myo-Inositol	65.11 ± 67.42	32.10 ± 41.63	0.15
Proline	61.16 ± 42.52	54.98 ± 30.32	0.71
Propionate	259.00 ± 197.97	271.85 ± 125.52	0.82
Pyroglutamate	46.73 ± 62.22	53.45 ± 55.10	0.48
Serine	79.21 ± 81.02	125.95 ± 109.38	0.11
Sucrose	114.01 ± 198.70	257.95 ± 505.73	0.38
Taurine	114.01 ± 59.95	133.11 ± 45.92	0.43
Threonine	51.18 ± 61.92	34.51 ± 42.36	0.48
trans-Aconitate	23.72 ± 12.24	18.58 ± 31.24	0.64
Trigonelline	5.03 ± 1.48	6.16 ± 5.50	0.46
Trimethylamine	2.23 ± 0.90	1.98 ± 1.32	0.65
Uracil	28.11 ± 9.96	46.40 ± 34.84	0.13
Uridine	37.75 ± 16.11	36.63 ± 34.74	0.92
Urocanate	21.14 ± 9.59	36.28 ± 60.57	0.43
Valerate	8.44 ± 14.02	28.10 ± 69.93	0.37
Valine	28.75 ± 12.49	34.58 ± 14.09	0.16
